# A vaginal fibroepithelial stromal polyp: a case report with magnetic resonance images

**DOI:** 10.1259/bjrcr.20210189

**Published:** 2022-01-06

**Authors:** Naoko Ogura, Mieko Inagaki, Ritsuko Yasuda, Shigeki Yoshida, Tetsuo Maeda

**Affiliations:** 1 Department of Obstetrics and Gynecology, Chibune General Hospital, Osaka, Japan; 2 Department of Diagnostic Radiology, Chibune General Hospital, Osaka, Japan

## Abstract

A fibroepithelial stromal polyp is a benign soft tissue tumour that can occur in the vagina, vulva and uterine cervix. Magnetic resonance imaging (MRI) findings have been reported in patients with vulvar fibroepithelial stromal polyps, not in those with vaginal polyps. We present MRI findings of vaginal fibroepithelial stromal polyp in a postmenopausal female. A 1 to 2 cm firm vaginal mass arising from the left side of the vaginal wall with hypointense signal changes on T1W MRI was identified. A well-defined vaginal mass (1 cm diameter) was detected with inhomogeneous signal intensity on T2W images. However, a major portion had high signal intensity on diffusion-weighted images. A benign vaginal lesion with oedematous changes or myxoid degeneration was suspected. Vaginal resection was performed, and fibroepithelial stromal polyp was pathologically diagnosed. MRI may be a useful non-invasive modality for preoperatively diagnosing vaginal fibroepithelial stromal polyps.

## Clinical presentation

A 60-year-old female presented with abnormal vaginal bleeding. The patient never had remarkable medical history or vaginal bleeding after menopause before her presentation. She received no medications or supplements. Physical examination revealed a 1–2 cm firm vaginal mass originating from the left side of the vaginal wall. There was no ulceration of the vagina or vulva.

All routine laboratory test results were normal including cervical cytology and endometrial cytology.

## Differential diagnosis

Differential diagnoses were considered to include various vaginal masses, such as angiomyofibroblastoma, aggressive angiomyxoma and squamous cell carcinoma. These masses show low signals on T1W image and high signals on T2W MRI. Since these findings were similar to each other, it was difficult to make a differential diagnosis before treatment. However, contrast-enhanced imaging and/or DWI could help with diagnosis. Regarding angiomyofibroblastoma, contrast-enhanced imaging reports are rare and almost all of them report homogenous hyperenhancement.^1^ Enhancement of aggressive angiomyxoma is described as having an intense, swirled or layered pattern. In addition, aggressive angiomyxoma exhibits very high signal intensity on DWI and high intensity on ADC maps, suggesting a T2 shine-through effect.^2^ This finding is similar to that of the current case. Aggressive angiomyxoma has the characteristics of large size (usually greater than 10 cm). In our case, the tumour size was highly variable and ranged from 1 to 60 cm.^3^ Thus, aggressive angiomyxoma demonstrating a small lesion such as in our case may be very rare. The MRI features of vaginal squamous cell carcinoma, changing in each stage, generally show low signals on T1W images and intermediate-to-high signals on T2W images relative to the submucosal and muscularis layers. Although the role of DWI in vaginal cancer is still unknown, it could be useful for suspecting the lesion to be malignant.^4^

## Investigations

Macroscopic findings could not exclude the possibility of aggressive angiomyxoma on a malignant tumour. We performed transvaginal ultrasonography. However, the mass had indistinct margins and we could not identify it clearly. We did not perform biopsy: the tumour was too small to perform biopsy that could have missed the exact area of tissue. Thus, routine pelvic magnetic resonance imaging (MRI) was performed.

**Figure 1.**
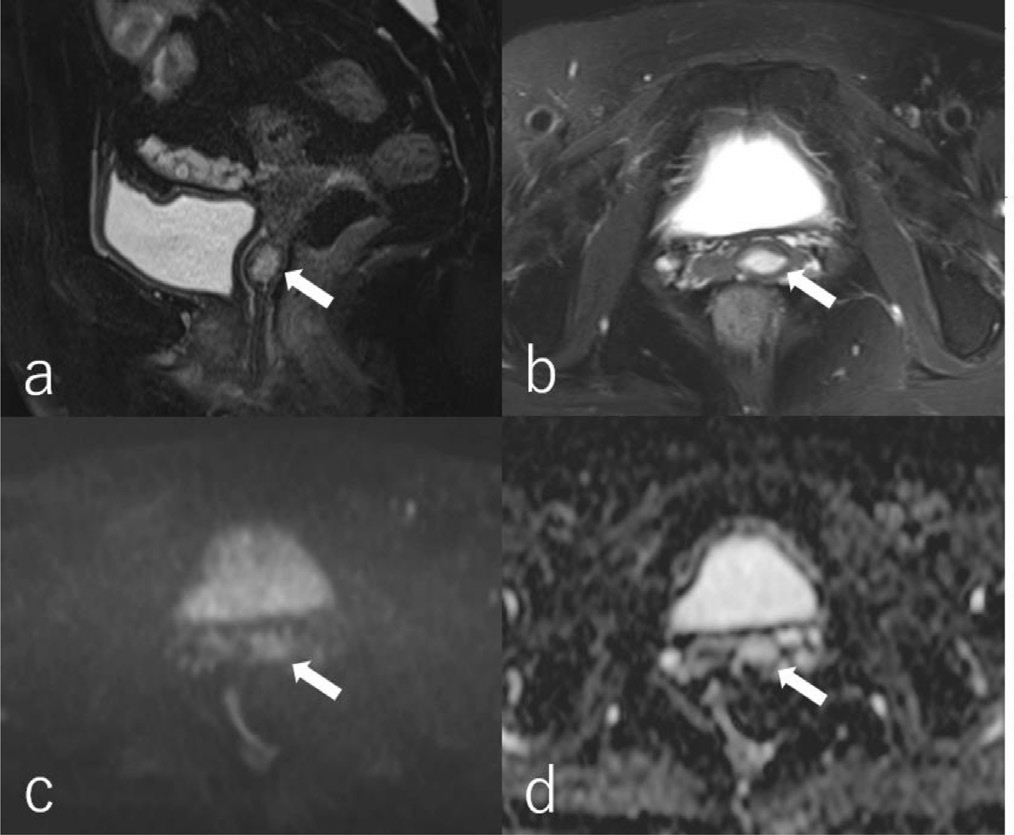
Magnetic resonance (MR) images in a 60-year-old female with vaginal fibroepithelial stromal polyp. Fat-suppressed T2 sagittal image (**a**)and axial image (**b**)show a well-defined vaginal mass (1 cm in diameter) with an inhomogeneous signal intensity (arrow). A MR diffusion-weighted image (b-value = 800 s/mm^2^) (**c**)shows a major portion of the mass with high intensity (arrow). A MR apparent diffusion coefficient (ADC) map (**d**)shows that the mean ADC value of the mass is relatively high (2.5 × 10^–3^ mm^2^/s) (arrow). All MR images are acquired using a 3 T Magnetom Skyra (Simens Healthcare, Erlangen. Germany).

T2W MRI showed a small nodular lesion, which was isointense relative to the vaginal wall and myometrium, in the upper site of the vaginal cavity. On T2W MRI, a well-defined vaginal lesion (1 cm diameter) showed inhomogeneous hyperintense relative to the surrounding tissue.

(Figure 1a and b). Furthermore, a major portion of the mass showed high signal intensity on diffusion-weighted images (DWI) with a b-value of 800 s/mm^2^. However, since the mean apparent diffusion coefficient (ADC) value of the lesion was relatively high (2.1 × 10^–3^ mm^2^/s), this high intensity was considered a T2 shine-through effect (Figure 1c and d). Thus, a benign vaginal lesion with oedematous changes or myxoid degeneration was suspected.

## Treatment

Vaginal tumour resection was performed for establishing definitive diagnosis and treatment. Since the MRI findings indicated that the tumour was likely benign, it was excised with an electric scalpel at the root of the stalk without taking a radical surgical margin.

## Outcome

**Figure 2.**
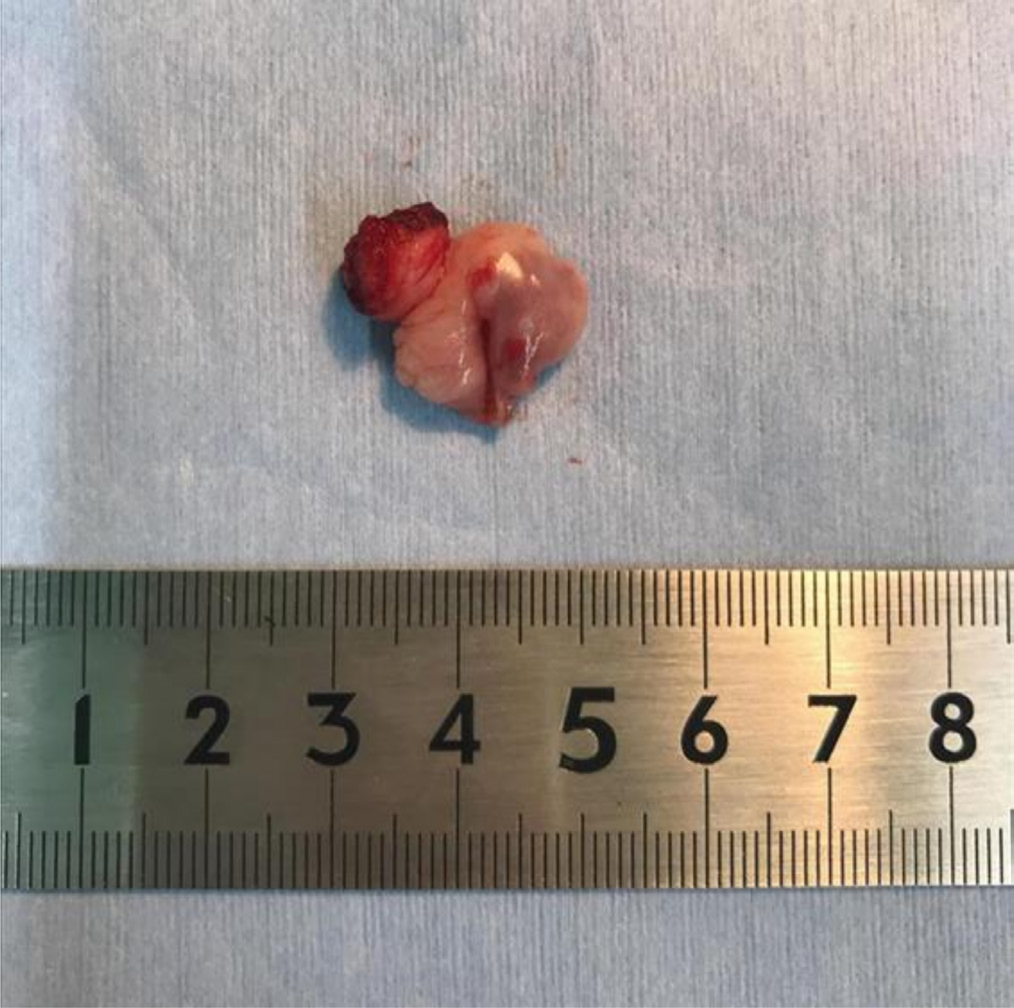
Macroscopic pathology of a vaginal fibroepithelial stromal polyp from a 60-year-old female. The 11–12 o'clock direction of the pathological specimen is the resection edge.

**Figure 3.**
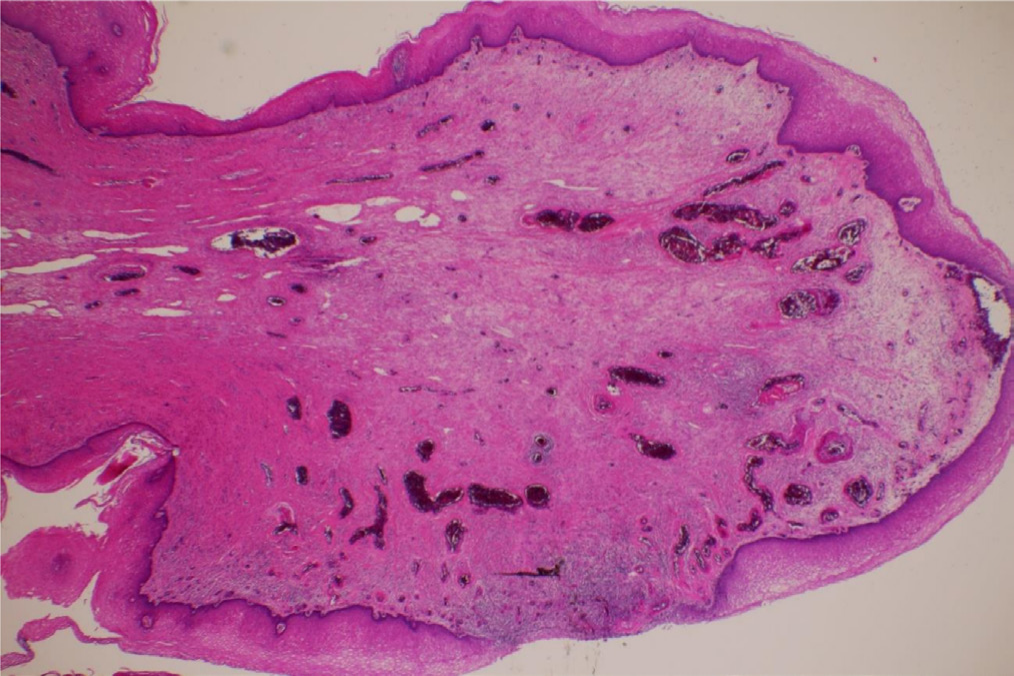
Pathology of the resected specimen (haematoxylin and eosin stain,×100) acquired from a 60-year-old female with vaginal fibroepithelial stromal polyp. The histology of the resected tumour reveals the presence of fibrosis and a partially oedematous lesion without atypia.

Macroscopic findings identified a 1.5 cm smooth mass (Figure 2), and pathological analysis of the resected tumour tissue revealed the presence of fibrosis and a partially oedematous lesion without atypia (Figure 3). Thus, the patient was diagnosed with fibroepithelial stromal polyp (FESP).

## Follow-up

Since the follow-up and postoperative course were uneventful for 2 years, we performed only visual inspection of the surgical site.

## Discussion

FESP is a benign soft tissue tumour that can occur in the vagina, vulva and uterine cervix. FESP is associated with oestrogen and progesterone in reproductive-aged females. FESP patients are commonly asymptomatic upon pelvic examination. However, sometimes this tumour can be found in females presenting with abnormal vaginal bleeding, lower abdominal discomfort and profuse vaginal discharge.^5–7^ Complete surgical excision is curative. However, incomplete resection may lead to disease recurrence.^8^

In the current case, rough diagnosis of this disease has been made on visual examination, palpation and ultrasonography, and final diagnosis was established by pathological examination. Typical pathological features of FESP are marked hypercellularity, marked cytologic pleomorphism, mitotic counts of more than ten mitoses per ten high-power fields, and atypical mitoses.^9^

To date, several cases of FESP arising from various organs including the vulva have been reported in the English-language literature. However, no MRI findings of vaginal FESP have ever been reported.

Although a contrast-enhanced MR study was not performed, this present case report is the first to describe MR features of this rare tumour. The current case revealed non-specific findings on T1W and T2W imaging. However, DWI findings were not suggestive of malignancy. It is well known that DWI-MRI is an important method for differentiating benign tumours from malignant ones, including gynaecological lesions. DWI-MRI measures the Brownian motion of molecules and highlights the increased cellularity of cancer tissue through quantitative evaluation using ADC mapping. Therefore, MRI might be useful for preoperatively diagnosing vaginal tumours, as in this case.

Differential diagnoses of other vaginal masses include angiomyofibroblastoma, aggressive angiomyxoma and squamous cell carcinoma. However, pathological variability might cause misdiagnoses.^8^ Therefore, it is important to distinguish FESP from other malignant tumours, such as squamous cell carcinoma, to prevent unnecessary treatment.

To our best knowledge, we are the first to present the typical MRI features of FESP, which could help differentiate FESP from other vulvovaginal stromal tumours and may be useful for other cases.

## Learning points

- Fibroepithelial stromal polyp (FESP) is a benign soft tumour roughly diagnosed in reproductive-aged females by visual examination, palpation and ultrasonography and finally diagnosed by pathological examination.
- Magnetic resonance imaging (MRI) findings of vaginal FESP may be non-specific. However, MRI with diffusion-weighted images (DWIs) and the apparent diffusion coefficient (ADC) map may help preoperatively differentiate benign tumours from malignant ones, including gynaecological lesions.
